# Effect of the TIM-3/Gal-9 signaling pathway on macrophage polarization in peri-implantitis

**DOI:** 10.1371/journal.pone.0328258

**Published:** 2025-07-18

**Authors:** Lu-Jin Cheng, Shu-Ya Dong, Xiao-Feng Ni, Long Mei, Jun-Gang He, Aynur Mamat, Zhong-Cheng Gong

**Affiliations:** 1 Department of Oral Rehabilitation and Implantology, The First Affiliated Hospital of Xinjiang Medical University (Affiliated Stomatology Hospital), Urumqi, Xinjiang, People's Republic of China; 2 Stomatological Research Institute of Xinjiang Uygur Autonomous Region, Urumqi, Xinjiang, People's Republic of China; 3 Department of Stomatology, Shache County People’s Hospital, Kashgar Prefecture, People's Republic of China; 4 Oncological Department of Oral and Maxillofacial Surgery, The First Affiliated Hospital of Xinjiang Medical University (Affiliated Stomatology Hospital), Urumqi, Xinjiang, People's Republic of China; Rutgers: Rutgers The State University of New Jersey, UNITED STATES OF AMERICA

## Abstract

**Introduction:**

Peri-implantitis (PI) is a common complication of oral implant surgeries. Understanding the role of T-cell immunoglobulin and mucin domain-containing protein 3 (TIM-3) and its ligand galectin-9 (Gal-9) in PI is essential for advancing treatment strategies. This study investigated the effect of the TIM-3/Gal-9 signaling pathway on macrophage polarization in PI.

**Methods:**

We included 31 PI patients and 30 controls with healthy implants. Peripheral blood and peri-implant tissue samples were collected. Serum Gal-9 and cytokine levels were measured using ELISA. CD86, CD206, and TIM-3 expressions were analyzed via immunohistochemistry. Lipopolysaccharide was used to induce a PI environment in cells, which were divided into blank control, control, and Gal-9 groups. Flow cytometry detected M1, M2, TIM-3^+^M1, and TIM-3^+^M2 proportions.

**Results:**

There were positive expressions of CD86 and CD206 in peri-implant tissues of PI patients, indicating the presence of both macrophage phenotypes, with a notable predominance of M1. The proportions of CD86^+^M1 macrophages and CD206^+^M2 macrophages in peripheral blood were significantly elevated in the PI group, resulting in an increased M1/M2 ratio in the PI group. Correlation analyses indicated that both M1 and M1/M2 were positively correlated with the modified plaque index, modified sulcular bleeding index, and probing depth, suggesting that the M1/M2 ratio reflects the clinical severity of PI. In vitro experiments showed that the addition of Gal-9 led to a significant increase in the proportion of TIM-3^+^M1 and TIM-3^+^M2 macrophages and a decrease in M1 cell proportions and M1/M2 ratio. The Gal-9 group exhibited significantly reduced levels of pro-inflammatory cytokines IL-1β and TNF-α. A strong negative correlation was found between TNF-α levels and TIM-3^+^M1 macrophages. However, no significant difference was found in the anti-inflammatory cytokine IL-10 between the control and Gal-9 groups.

**Conclusion:**

The TIM-3/Gal-9 signaling pathway plays a crucial role in modulating macrophage polarization in PI. This work may provide evidence for the development of novel therapeutic targets for managing PI.

## Introduction

Peri-implantitis (PI) is a major complication of oral implant surgery, which can easily lead to decreased periodontal attachment, bone tissue absorption, and even implant loosening, detachment, or failure, causing significant physical and mental distress to patients [1,2]. Up to now, there is still no consensus on the etiology and prevention strategies of PI, posing a critical barrier to the long-term success of oral implants and presenting a complex challenge in clinical management. Although implantation surgeries may be successful, implants can still develop PI, potentially due to local infections. It has been suggested that infections occurring after implant placement may disrupt the balance of the host microbiome, subsequently affecting the host immune response [3]. The inflammation caused by immune imbalance can negatively impact implant success rates [4]. Lipopolysaccharide (LPS), as one of the key initiators of PI [5], can activate various immune cells through cellular signaling. Among these, macrophages (Mφ) are crucial immune cells involved in inflammatory and repair responses in PI [6]. The secretion of pro-inflammatory cytokines and tissue repair factors characterizes the external phenotype of Mφ, while changes in metabolic pathways represent their internal characteristics. These changes are of significant importance for regulating the inflammatory process and tissue repair [7]. Mφ can polarize into M1 and M2 subtypes. M1 promotes inflammatory responses while M2 inhibits excessive inflammation. An imbalance between these subtypes can lead to disease progression in different directions [8]. Moreover, these two Mφ phenotypes and their metabolic states, regulated by various cytokines and signaling pathways, maintain a dynamic balance. This balance directly influences the development of PI [6,9].

The T-cell immunoglobulin and mucin domain-containing protein 3 (TIM-3) is an important regulatory molecule in the Mφ polarization [10,11]. Its ligand, galectin-9 (Gal-9) is expressed in the nucleus, cell surface, and cytoplasm, and is present in nearly all biological systems [12]. Gal-9 is considered a novel target for immune regulation and plays different roles in various inflammatory diseases through its interaction with TIM-3 [13]. However, to date, there have been no studies investigating the regulation of Mφ polarization phenotypes and internal metabolic pathways by the TIM-3/Gal-9 axis in PI.

Here, this study examined the expression of TIM-3, Gal-9, M1, and M2 in the local tissues and peripheral blood of PI patients, as well as their correlation with clinical parameters. Simultaneously, we constructed a cellular model of PI to analyze the polarization of Mφ, inflammatory factors, and internal metabolism concerning PI. Furthermore, we explored the correlation between TIM-3 expression, Mφ polarization phenotypes, and metabolism through the exogenous supplementation of Gal-9. Our findings may provide new immunological insights for the prevention and treatment of PI.

## Materials and methods

### Study participants

The patients who received implant treatment at the Department of Oral Rehabilitation and Implantology of the First Affiliated Hospital of Xinjiang Medical University between 10/03/2022 and 31/12/2023 were selected as the study participants. A total of 31 patients with PI were finally included in the PI group, while 30 healthy implant patients without PI served as the control group. Peripheral blood samples were collected from both the PI group and the control group, and peri-implant tissues were obtained from 16 patients in the PI group. The diagnosis of PI was based on the classification established at the World Workshop on Periodontal and Peri-implant Diseases and Conditions organized by the American Academy of Periodontology and the European Federation of Periodontology in 2017 [14]. The criteria included mild bleeding on probing with/without suppuration, probing depth (PD) ≥6 mm, and radiographic marginal bone loss (i.e., minimum 3 mm of bone loss from the dental implant coronal part at the most proximal aspect). Inclusion criteria: 1) Patients who had signed and provided informed consent; 2) Aged between 18 and 65 years old; and 3) Patients with a single missing tooth. Exclusion criteria: 1) Patients with general contraindications in dental and surgical treatment; 2) Patients with untreated periodontal disease; 3) Pregnant or lactating women; 4) Patients with autoimmune and/or inflammatory diseases; 5) Patients with uncontrolled diabetes (HbA1c>7); 6) Patients receiving corticosteroid therapy; and 7) Smokers. Relevant patient demographics, including age, sex, and medical history, were collected and analyzed to control for potential confounding factors. The work described has been carried out in accordance with the World Medical Association Declaration of Helsinki for experiments involving humans. The collection of clinical specimens and laboratory examinations of tissue specimens strictly followed the ethical regulations. This study was approved by the Ethics Committee of the First Affiliated Hospital of Xinjiang Medical University (Approval No.: 20220308-147). Written informed consent was obtained from all patients.

### Clinical examination

The modified plaque index (mPLI) was scored as follows: 0 = No plaque; 1 = Plaque detectable with light scraping on the surface of the implant; 2 = Plaque visible to the naked eye; 3 = Abundant soft matter. The modified sulcular bleeding index (mSBI) was evaluated as 0 = No bleeding upon probing; 1 = Point bleeding in the gingival sulcus; 2 = Linear bleeding in the gingival sulcus; 3 = Spontaneous bleeding. PD was measured using a resin periodontal probe by assessing the depth from the gingival margin to the base of the implant sulcus at the buccal and lingual sides near the mesial, central, and distal areas, along the longitudinal axis of the implant, followed by calculating the average of the depths at six positions [15]. The clinical score was determined as the sum of the mPLI, mSBI, and PD.

### ELISA

The levels of tumor necrosis factor-alpha (TNF-α), interleukin-1 beta (IL-1β), and IL-10 in cell culture supernatant, as well as Gal-9 in serum, were detected using ELISA kits (Jianglaibio, Shanghai, China), following the kit instructions. The optical density values at a wavelength of 450 nm were measured. The concentrations of Gal-9, TNF-α, and IL-1β were calculated based on the standard curve.

### Immunohistochemistry

The peri-implant tissues were fixed in formalin for 24 h, embedded in paraffin, sliced into sections (6 μm thickness), deparaffinized, rehydrated, and treated with citrate buffer. The slides were then incubated overnight at 4°C with primary antibodies: anti-CD86 (1:50, Boster, Wuhan, China), anti-CD206 (1:200, Boster, China), anti-TIM-3 (1:1000, Abcam, Cambridge, UK), followed by incubation with HRP-conjugated secondary antibodies (Proteintech, Wuhan, China) for 1 h. DAB chromogen was used for visualization, and three random fields were selected for observation under a microscope. The expression level was scored based on the staining intensity (0–3 points for negative staining, light yellow, light brown, and dark brown) and the positive staining percentage (1–4 points for 0–25%, 26–50%, 51–75%, and > 76%). The scoring was conducted by two experienced senior physicians.

### Immunofluorescence staining

The peri-implant tissue sections were subjected to antigen retrieval, followed by blocking with 3% bovine serum albumin for 30 min. Subsequently, the sections were incubated with primary antibodies of anti-CD68 (1:3000, Proteintech) and anti-TIM-3 (1:50, Sabbiotech, Nanjing, China) overnight at 4°C. After washing, the incubation with secondary antibodies of CY3 labeled goat anti-rabbit IgG (1:300, Servicebio, Wuhan, China) and Alexa Fluor 488 labeled goat anti-mouse IgG (1:300, Servicebio) was conducted at room temperature for 50 min in the dark. The cell nuclei were stained with DAPI at room temperature for 10 min in the dark. After fluorescent quenching, the sections were mounted with an anti-fluorescence quenching mounting medium and immediately observed and photographed under a fluorescence microscope. Two experienced pathologists evaluated the immunofluorescence staining image**s**.

### Cell treatment

THP-1 cells (Procell, Wuhan, China) were seeded into a 6-well plate, with each well containing 1 x 10^6^ cells. The cells were cultured with a complete culture medium containing 100 ng/mL phorbol 12-myristate 13-acetate (MCE, Shanghai, China) for 24 h to induce M0 cells. Then, the M0 cells in the control group were treated with 100 ng/mL LPS (Solarbio, Beijing, China), and 20 ng/mL interferon-gamma (MCE, Shanghai, China), for 24 h [16]. The M0 cells in the Gal-9 group were treated with 100 ng/mL LPS, 20 ng/mL interferon-gamma, and 1000 ng/mL Gal-9 (ACRO, USA) for 24 h. THP-1 cells without induction or treatment served as the blank control group. After 24 h of culture, the cells in each group were collected for flow cytometry analysis.

### Flow cytometry

The anticoagulated peripheral blood (100 μL) was incubated with the antibodies against CD68-PECY7 (Biolegend, San Diego, CA, USA), CD14-PECF594 (BD, Franklin Lake, NJ, USA), CD206-BB700 (BD, USA), CD86-AF488 (Biolegend, USA), and TIM-3-PE (BD, USA) at a volume of 5 μL each. The incubation was performed at room temperature in the absence of light for 15 min. Subsequently, the blood samples were subjected to red blood cell lysis. On the other hand, the cells were collected from each group and incubated with antibodies against CD14 (Biolegend, USA), CD86 (Biolegend, USA), CD206 (BD, USA), and TIM-3 (BD, USA) at 4°C in the dark for 30 min. After washing with PBS, the blood and cell samples were subjected to flow cytometry analysis on BD AriaII (BD, USA). The proportions of M1, M2, TIM-3^+^M1, and TIM-3^+^M2 were then analyzed using FlowJo10.8 software.

### Measurement of pH value

The supernatants from both the Gal-9 group and the control group were collected into 1.5 ml EP tubes. A glass rod was used to transfer a drop of the cell supernatant onto precision pH test paper. The color of the test paper was then compared with a standard color chart to determine the pH, thereby assessing the acidity and alkalinity of the cell supernatant.

### Statistical analysis

SPSS 25.0 (Chicago, IL, USA) was used for data analysis. Continuous variables that conformed to a normal distribution and demonstrated homogeneity of variance are represented as mean ± standard deviation. Independent samples t-tests were used to evaluate statistical differences between two groups, while comparisons among multiple groups were conducted using one-way analysis of variance and Tukey’s test. Categorical variables, which are represented by number and percentage, were compared with the chi-square test. Correlation analyses were performed through linear regression and Spearman correlation analysis. A p-value of less than 0.05 was considered statistically significant.

## Results

### Baseline clinical data

The baseline clinical data are presented in Table 1. There were 31 cases in the PI group, with 22 males (71.0%) and 9 females (29.0%); and 30 cases in the control group, with 16 males (63.6%) and 14 females (36.4%). There were no statistically significant differences in gender and age between the two groups (P > 0.05). However, the PI group had significantly higher levels of mPLI, mSBI, and PD than the control group (P < 0.05).

**Table 1:** Baseline clinical data.

Grouping	PI group (n = 31)	Control group (n = 30)	t/χ^2^	P value
**Male (%)**	22 (71.0)	16 (53.33)	2.02	0.155
**Female (%)**	9 (29.0)	14 (46.67)		
**Age (years)**	47.61 ± 12.89	43.23 ± 9.76	1.49	0.137
**mPLI**	2.00 ± 0.83	2.00 ± 0.82	3.89	0.003
**mSBI**	2.00 ± 0.89	1.00 ± 0.82	4.67	<0.001
**PD (mm)**	5.00 ± 1.83	3.00 ± 0.71	5.59	<0.001

Note: PI, peri-implantitis; mPLI, modified plaque index; mSBI, modified sulcular bleeding index; PD, probing depth.

### Analysis of CD86, CD206, and TIM-3 expression in peri-implant tissues of PI patients

**Fig 1:**
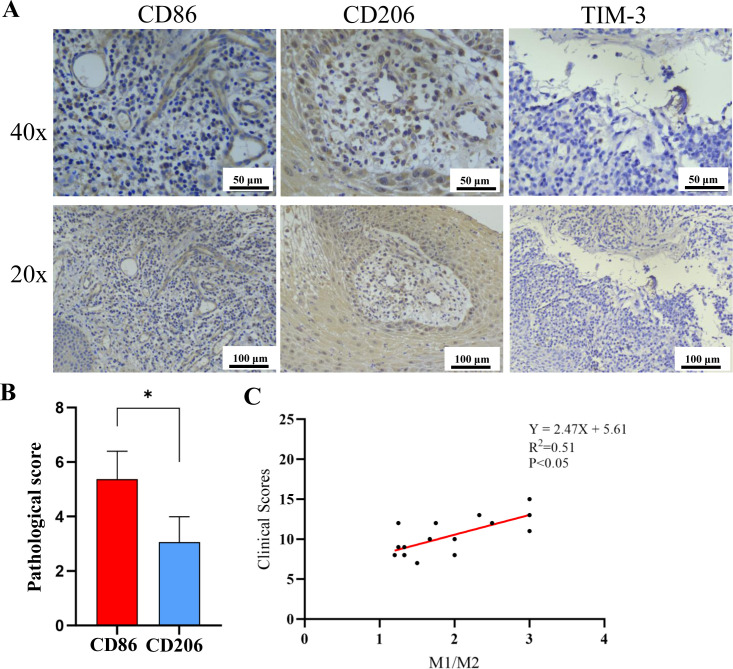
Immunohistochemical staining of CD86, CD206, and TIM-3. A: Representative immunohistochemical staining results of CD86, CD206, and TIM-3 in peri-implant tissues of PI patients with magnifications of 20x (scale bar: 100 μm) and 40x (scale bar: 50 μm). B: The pathological scores of CD86 and CD206. *p < 0.05. C: Linear regression analysis of M1/M2 and clinical scores.

The expressions of CD86, CD206, and TIM-3 in peri-implant tissues of PI patients were evaluated with immunohistochemistry. The representative immunohistochemical staining results are shown in Fig 1A. There were positive expressions of CD86 and CD206 in peri-implant tissues of the PI group, indicating the presence of CD86^+^M1 and CD206^+^M2. Similarly, TIM-3 positive expression was also observed in peri-implant tissues of the PI group. However, it remains unclear whether they were TIM-3^+^ Mφ. The pathological scores of CD86 (5.38 ± 1.02) were significantly higher than those of CD206 (3.06 ± 0.93) (P < 0.05) (Fig 1B). Linear regression analysis demonstrated a significant positive correlation between the M1/M2 ratio in peripheral blood and clinical scores (R^2^ = 0.51; P < 0.05) (Fig 1C).

### Immunofluorescence staining analysis of CD68^+^ Mφ and CD68^+^TIM-3^+^ Mφ in peri-implant tissues of PI patients

**Fig 2:**
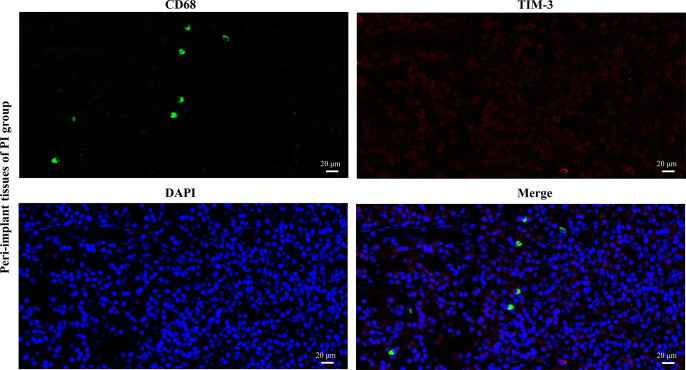
Representative immunofluorescence images of CD68 and TIM-3. The expression of CD68 and TIM-3 in the peri-implant tissues was stained with immunofluorescence (scale bar: 20 μm).

The results of immunofluorescence staining revealed that CD68^+^ Mφ (green fluorescence), TIM-3^+^ cells (red fluorescence), and CD68^+^TIM-3^+^ Mφ (merged) were present in the peri-implant tissues of PI patients (Fig 2). However, their numbers were relatively low.

### Analysis of CD86^+^M1 and CD206^+^M2 proportions in peripheral blood

**Fig 3:**
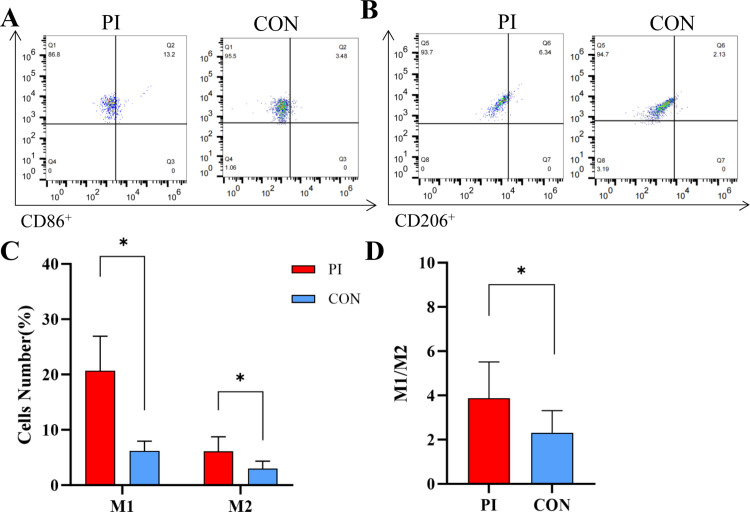
Analysis of CD86^+^M1 and CD206^+^M2 proportions in peripheral blood. Flow cytometry was performed to detect cell proportion. A: The gating strategy for CD86^+^M1 in the PI and control groups. B: The gating strategy for CD206^+^M2 in the PI and control groups. C: Comparison of CD86^+^M1 and CD206^+^M2 proportions between the PI and control groups. D: Comparison of M1/M2 ratio between the PI and control groups. *p < 0.05.

To assess the Mφ populations in peripheral blood, flow cytometry was performed. The results showed that in the PI group, the proportions of CD86^+^M1 (20.69 ± 6.23) and CD206^+^M2 (6.17 ± 1.79) were significantly higher than that of the control group (CD86^+^M1: 6.10 ± 2.63 and CD206^+^M2: 3.02 ± 1.32) (Fig 3A–3C). Within the P1 group, the proportion of M1 was significantly higher than that of M2 (P < 0.05). Additionally, the PI group had a significantly elevated M1/M2 ratio than the control group (3.88 ± 1.64 vs 2.32 ± 1.00) (P < 0.05) (Fig 3D). This data indicates that there are increased CD86^+^M1 proportions and M1/M2 ratios in PI patients.

### Correlation analysis among M1 and M1/M2 in peripheral blood with mPLI, mSBI, and PD

Spearman correlation analysis revealed that M1 and M1/M2 in peripheral blood were positively correlated with mPLI, mSBI, and PD (P < 0.01) (Table 2). Among them, M1/M2 had a higher correlation with mPLI, mSBI, and PD. There was a positive correlation among mPLI, mSBI, and PD (P < 0.01). Notably, there was a strong correlation between mPLI and mSBI (R = 0.931).

**Table 2:** Spearman correlation analysis among M1 and M1/M2 in peripheral blood with mPLI, mSBI, and PD.

Variables		M1	M2	M1/M2	mPLI	mSBI	PD
**M1**	R	1					
	*P*						
**M2**	R	0.773	1				
	*P*	<0.01					
**M1/M2**	R	0.520	−0.079	1			
	*P*	<0.01	0.544				
**mPLI**	R	0.432	0.055	0.622	1		
	*P*	0.001	0.676	<0.01			
**mSBI**	R	0.470	0.094	0.637	0.931	1	
	*P*	<0.01	0.473	<0.01	<0.01		
**PD**	R	0.566	0.139	0.758	0.425	0.499	1
	*P*	<0.01	0.287	<0.01	<0.01	<0.01	

Note: mPLI, modified plaque index; mSBI, modified sulcular bleeding index; PD, probing depth.

### Linear regression analysis of M1, M1/M2, clinical score, serum Gal-9, and TIM-3^+^ Mφ

**Fig 4:**
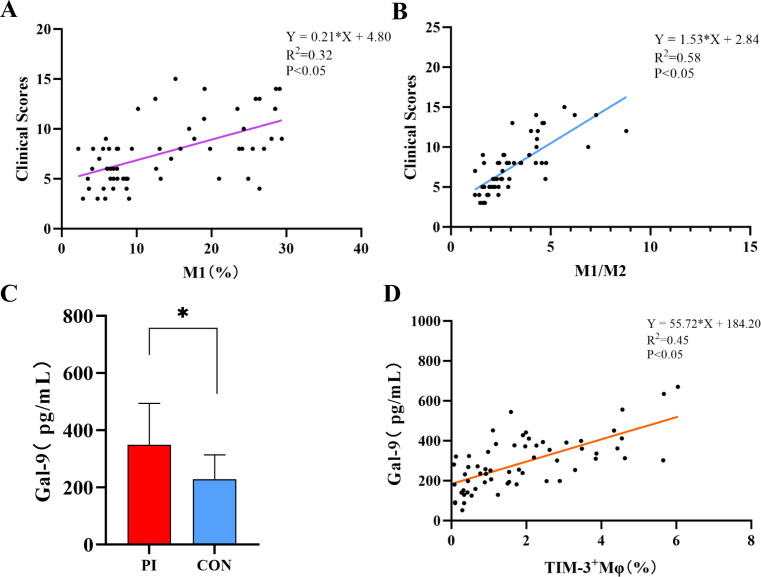
Linear regression analysis. A: Linear regression analysis of M1 in peripheral blood and clinical score. B: Linear regression analysis of M1/M2 in peripheral blood and clinical score. C: Serum Gal-9 level as assessed by ELISA. *p < 0.05. D: Linear regression analysis of TIM-3^+^ Mφ in peripheral blood and serum Gal-9.

The results of linear regression analysis indicated that M1 had a low correlation with the clinical score (R^2^ = 0.32, P < 0.05) (Fig 4A). There was a correlation between M1/M2 and the clinical score (R^2^ = 0.58, P < 0.05) (Fig 4B). ELISA revealed that the level of Gal-9 in the peripheral blood of the PI group (349.19 ± 144.25 pg/mL) was significantly higher than that in the control group (229.24 ± 84.67 pg/mL) (P < 0.02) (Fig 4C). Additionally, there was a correlation between TIM-3^+^ Mφ and Gal-9 (R^2^ = 0.45, P < 0.05) (Fig 4D).

### Analysis of CD86^+^M1, TIM-3^+^M1, CD206^+^M2, and TIM-3^+^M2 proportions and M1/M2 ratio *in vitro*

**Fig 5:**
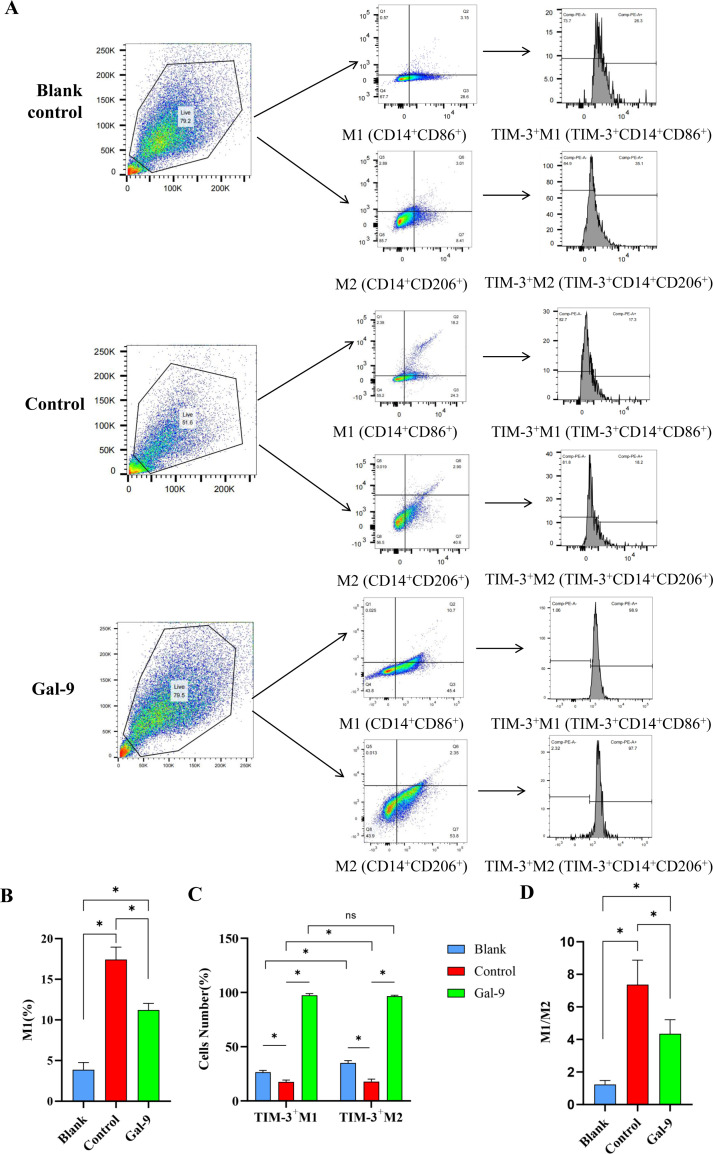
Analysis of CD86^+^M1, TIM-3^+^M1, CD206^+^M2, and TIM-3^+^M2 proportions and M1/M2 ratio in vitro. A: Gating strategies of CD86^+^M1, TIM-3^+^M1, CD206^+^M2, and TIM-3^+^M2 in the blank control, control, and Gal-9 groups. B: Proportions of CD86^+^M1 in each group. C: Proportions of TIM-3^+^M1 and TIM-3^+^M2 in each group. D: The ratio of M1/M2 in each group. *p < 0.05.

Flow cytometry analysis of cells revealed that there were significant differences in the proportion of CD86^+^M1 cells among the three groups (P < 0.05). Specifically, the proportion of CD86^+^M1 cells (Fig 5A and 5B) and the ratio of M1/M2 (Fig 5D) in the control group were significantly higher than those in the blank control and Gal-9 groups (P < 0.05); and those in the Gal-9 group was also significantly higher than that in the blank control group (P < 0.05). There was no significant difference in CD206^+^M2 among the three groups (P > 0.05) (data not shown). Furthermore, the proportions of TIM-3^+^M1 cells and TIM-3^+^M2 cells among the three groups were also significantly different (P < 0.05) (Fig 5A and 5C). In particular, the Gal-9 group had a significantly higher proportion of TIM-3^+^M1 cells and TIM-3^+^M2 cells than the control group (P < 0.05), while their proportions in the blank control group were significantly higher than those in the control group (P < 0.05). However, no significant difference was found between TIM-3^+^M1 and TIM-3^+^M2 in the Gal-9 group (P > 0.05).

### Analysis of cytokine expression and correlation in the cell culture supernatant

**Fig 6:**
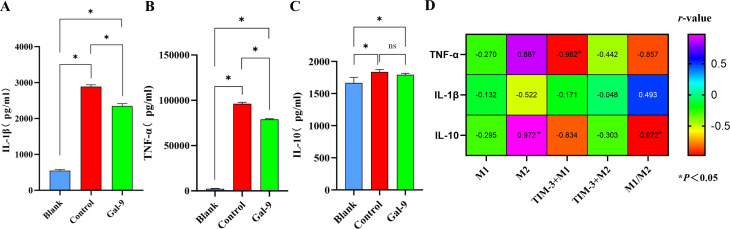
Analysis of cytokine expression and correlation in the cell culture supernatant. Cytokine levels in the culture supernatant were detected with ELISA. A: Level of IL-1β. B: Level of TNF-α. C: Level of IL-10. D: Correlation analysis of TNF-α, IL-1β, and IL-10 with M1, M2, TIM-3^+^M1, TIM-3^+^M2, and M1/M2 ratio. *p < 0.05.

We detected the cytokine levels in the culture supernatant by using ELISA. The results revealed that the levels of the pro-inflammatory cytokine IL-1β (Fig 6A) and TNF-α (Fig 6B) in the Gal-9 group were significantly lower than those in the control group (P < 0.05). However, there was no significant difference in the anti-inflammatory cytokine IL-10 between the control and Gal-9 groups (P > 0.05) (Fig 6C). Further analysis of the correlation between cytokine levels and M1, M2, TIM-3^+^M1, TIM-3^+^M2, and M1/M2 ratio revealed a strong negative correlation between TNF-α and TIM-3^+^M1 (P < 0.05) (Fig 6D). Additionally, a positive correlation was observed between IL-10 and M2, while a negative correlation was found with the M1/M2 ratio (P < 0.05) (Fig 6D).

### Analysis of pH values in the culture supernatants

**Fig 7:**
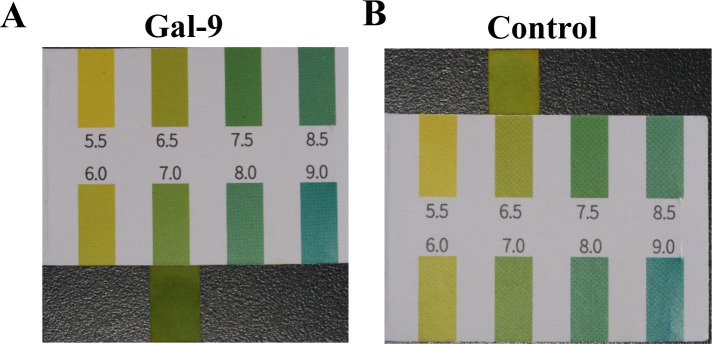
Analysis of pH values in the culture supernatants. A: The pH value of the Gal-9 group. B: The pH value of the control group.

The pH values of cell supernatants from the Gal-9 group and the control group were measured. As shown in Fig 7A, the pH value of the Gal-9 group supernatant was 7.0, whereas Fig 7B indicated that the pH value of the control group supernatant was 6.5.

## Discussion

LPS in the PI microenvironment may induce Mφ polarization and alter their internal metabolism [17,18]. TIM-3 serves as a crucial regulatory molecule for Mφ polarization and metabolism, while Gal-9 acts as a ligand that modulates Mφ functions and maintains immune balance by binding to TIM-3 [19,20].

In our study, we conducted immunohistochemistry on the peri-implant tissues in PI patients. The results suggest that Mφ may polarize into M1 and M2 subtypes during PI, with the M1 level higher than the M2 level in local tissues. This observation is consistent with previous findings, which reported a significant presence of M1 in PI samples [9,21]. We performed a linear regression analysis of the M1/M2 ratio in peri-implant tissues and the clinical score, which revealed a correlation. This interesting finding warrants further exploration of the underlying relationship. Furthermore, we detected TIM-3 expression on Mφ in the peri-implant tissues of PI patients and peripheral blood. However, the expression levels of TIM-3 were low, consistent with the study by Sun et al, showing that TIM-3 expression decreased during inflammation, leading Mφ to polarize toward M1 [22]. Additionally, we detected Gal-9 in the serum of PI patients for the first time through ELISA in this study. Based on these findings, we conducted a linear regression analysis between TIM-3^+^Mφ and Gal-9, which showed a correlation. This suggests that the Gal-9/TIM-3 signaling pathway in PI patients may regulate Mφ polarization and affect the onset and progression of PI, although the specific regulatory mechanisms remain unclear.

The association between oral inflammatory diseases and systemic diseases has been confirmed [23]. The imbalance of the M1/M2 ratio serves as a significant marker for inflammatory diseases [24,25]. Accordingly, this study innovatively assessed the proportions of Mφ, M1, and M2 in the peripheral blood of PI patients. Our results found that the polarization of M1 was significantly elevated in the peripheral blood of PI patients, resulting in an imbalance of the M1/M2 ratio. A linear regression analysis of the M1/M2 ratio in peripheral blood and clinical score identified a correlation between the two. This intriguing phenomenon further suggests that the M1/M2 ratio may reflect the clinical severity of PI from a specific perspective. Therefore, the measurement of the M1/M2 ratio in peripheral blood may be used as a straightforward and effective method to indicate the progression of PI and to regulate changes in M1/M2, thereby offering new strategies for the prevention and treatment of PI.

To further understand the effects of TIM-3, Gal-9, and Mφ polarization phenotypes in PI, we constructed a PI cell model in vitro and induced Mφ polarization to M1. Our results found that the TIM-3^+^M1 proportion was relatively low in the control group, which is consistent with the findings by Zhang et al [11]. Moreover, when pro-inflammatory factors IL-1β and TNF-α were elevated, the levels of M1 cells also increased. Thus, we speculate that the expression levels of pro-inflammatory factors may have some correlation with the Mφ polarization phenotype. Additionally, Lv et al reported that Gal-9 inhibited M1 polarization and reduced the M1/M2 ratio [26]. Consistently, we found that when Gal-9 was exogenously added as an intervention, the TIM-3^+^M1 and TIM-3^+^M2 proportions increased, while the M1 cell proportion decreased, leading to a lower M1/M2 ratio. This finding implies that Gal-9 may play a role in promoting Mφ polarization towards the M2 phenotype.

Furthermore, our study found reduced levels of TNF-α and IL-1β in the Gal-9 group, yet it remains unclear whether this reduction is a direct outcome of TIM-3 activation or a secondary effect. This question warrants further examination. Additionally, we observed another interesting phenomenon: the pH of the supernatant in the control group was acidic, while that in the Gal-9 group was neutral. Given the changes in pro-inflammatory factors, this provides a further rationale to speculate that there may be some association between the expression levels of pro-inflammatory factors and Mφ polarization and metabolism. Are there associations between Gal-9, TIM-3, pro-inflammatory factors, and the internal metabolism and polarization phenotype of Mφ? This warrants further consideration. It has been shown that the expression of IL-1β and TNF-α enhances Mφ glycolysis, leading to the expression of its pro-inflammatory phenotype [27]. Our study also found a strong negative correlation between TNF-α and TIM-3^+^M1, suggesting that TIM-3 may inhibit the production of TNF-α, consistent with a study by Zhang et al, which indicated that TIM-3 could inhibit the production of TNF-α and IL-1β, thereby suppressing Mφ glycolysis [28]. Interestingly, our analysis revealed that the anti-inflammatory cytokine IL-10 levels were comparable among the three groups, with no statistically significant difference observed between the control and the Gal-9 groups. This finding is consistent with a study conducted by Severino et al, which reported no difference in IL-10 levels between healthy and diseased implants [29]. Therefore, based on previous findings and our results, we propose a hypothesis that Gal-9 may activate TIM-3 and inhibit the production of pro-inflammatory factors such as TNF-α and IL-1β, potentially reducing the activity of Mφ glycolysis and altering its internal metabolic pathway, thereby affecting Mφ polarization.

Notably, due to ethical considerations and the critical role of health-associated peri-implant gingival tissue in maintaining implant success and preventing inflammation-related complications [30–32], we did not obtain peri-implant tissues from individuals with healthy implants. This is consistent with the study by Galarraga-Vinueza [21] et al, which similarly focused exclusively on PI patients. To address the absence of control data, we conducted flow cytometry analysis on peripheral blood samples from both PI patients and healthy implant controls. This approach validates our findings from PI peri-implant tissues while ensuring minimal impact on healthy individuals. Additionally, based on previous studies [33,34], we constructed an in vitro model simulating the PI environment by utilizing THP-1 cells exposed to LPS and IFN-γ. This model serves as a surrogate to evaluate the differences between PI and controls.

### Study limitations

Several limitations should be acknowledged. First, our in vitro findings may not fully recapitulate the complex in vivo environment of PI. Second, the sample size of our patient cohort is relatively small, which may limit the generalizability of our findings. Third, the direct mechanistic validation of TIM-3/Gal-9’s role in Mφ polarization is lacking. Future studies with in vivo models, diverse populations, or mechanistic validations are necessary to validate and extend our results regarding the role of TIM-3/Gal-9 in PI pathogenesis.

### Future directions

In future studies, it is essential to conduct knockdown experiments using siRNA, as well as overexpression studies utilizing CRISPR-Cas9. Additionally, Western blotting or qPCR should be employed to analyze critical signaling pathways, including NF-κB, STAT1/STAT6, PI3K/AKT, and MAPK. These methodologies are necessary to elucidate the mechanistic role of the TIM-3/Gal-9 signaling pathway in Mφ polarization within the context of PI. Moreover, we will utilize the Seahorse XF Analyzer for a comprehensive evaluation of metabolic reprogramming in M1 and M2 mediated by TIM-3/Gal-9 signaling. We will also conduct time-course experiments to capture temporal variations in cytokine production and TIM-3^+^M1 dynamics post-Gal-9 treatment. Furthermore, we intend to develop a co-culture system of Mφ and osteoclasts to investigate specific metabolic interactions within the PI immune microenvironment, aiming to connect immune dysregulation in PI with the metabolic interactions between these cell types. Additionally, given the critical role of TIM-3, which is highly expressed in exhausted T cells, future studies should focus on elucidating potential interactions between Mφ and T cells in the pathogenesis of PI, enhancing the understanding of the TIM-3/Gal-9 signaling axis and its impact on the inflammatory responses associated with PI. For therapeutic implications, future studies should evaluate the potential of immunomodulatory therapies that target the TIM-3/Gal-9 pathway to enhance implant success rates and improve patient outcomes in dental rehabilitation.

## Conclusion

The study presents compelling evidence for the regulatory effect of the TIM-3/Gal-9 axis on Mφ polarization and metabolism in PI. Elevated M1 polarization and an imbalance in Mφ phenotypes correlate with clinical severity in PI patients. By elucidating the relationship between TIM-3 and Gal-9 in regulating Mφ functions, this research proposes novel therapeutic targets for managing PI.

## References

[1] Salvi GE, Stähli A, Imber J, Sculean A, Roccuzzo A. Physiopathology of peri‐implant diseases. Clin Implant Dent Rel Res. 2022;25(4):629–39. doi: 10.1111/cid.13167 36515007

[2] Roccuzzo M, Mirra D, Roccuzzo A. Surgical treatment of peri-implantitis. Br Dent J. 2024;236(10):803–8. doi: 10.1038/s41415-024-7405-9 38789758 PMC11126382

[3] Alves CH, Russi KL, Rocha NC, Bastos F, Darrieux M, Parisotto TM, et al. Host-microbiome interactions regarding peri-implantitis and dental implant loss. J Transl Med. 2022;20(1):425. doi: 10.1186/s12967-022-03636-9 36138430 PMC9502891

[4] Alhamad M, Barão VA, Sukotjo C, Yerokhin A, Mathew MT. Unpredictable electrochemical processes in Ti dental implants: the role of Ti ions and inflammatory products. ACS Appl Bio Mater. 2023;6(9):3661–73. doi: 10.1021/acsabm.3c00235 37602778

[5] Pirih FQ, Hiyari S, Leung H-Y, Barroso ADV, Jorge ACA, Perussolo J, et al. A murine model of lipopolysaccharide-induced peri-implant mucositis and peri-implantitis. J Oral Implantol. 2015;41(5):e158-64. doi: 10.1563/aaid-joi-D-14-00068 24967609 PMC4391986

[6] Fretwurst T, Garaicoa-Pazmino C, Nelson K, Giannobile WV, Squarize CH, Larsson L, et al. Characterization of macrophages infiltrating peri-implantitis lesions. Clin Oral Implants Res. 2020;31(3):274–81. doi: 10.1111/clr.13568 31876318

[7] Al-Khami AA, Rodriguez PC, Ochoa AC. Energy metabolic pathways control the fate and function of myeloid immune cells. J Leukoc Biol. 2017;102(2):369–80. doi: 10.1189/jlb.1VMR1216-535R 28515225 PMC5505747

[8] Feito MJ, Casarrubios L, Oñaderra M, Gómez-Duro M, Arribas P, Polo-Montalvo A, et al. Response of RAW 264.7 and J774A.1 macrophages to particles and nanoparticles of a mesoporous bioactive glass: a comparative study. Colloids Surf B Biointerfaces. 2021;208:112110. doi: 10.1016/j.colsurfb.2021.112110 34555654

[9] de Waal YC, Eijsbouts HV, Winkel EG, van Winkelhoff AJ. Microbial characteristics of peri-implantitis: a case-control study. J Periodontol. 2017;88(2):209–17. doi: 10.1902/jop.2016.160231 27666672

[10] Nagahara K, Arikawa T, Oomizu S, Kontani K, Nobumoto A, Tateno H, et al. Galectin-9 increases Tim-3+ dendritic cells and CD8+ T cells and enhances antitumor immunity via Galectin-9-Tim-3 interactions. J Immunol. 2008;181(11):7660–9. doi: 10.4049/jimmunol.181.11.7660 19017954 PMC5886706

[11] Zhang W, Zhang Y, Fang Q. Effect of Galectin-9/Tim-3 pathway on the polarization of M1/M2 subtype in murine macrophages induced by lipopolysaccharide. Zhonghua Wei Zhong Bing Ji Jiu Yi Xue. 2018;30(9):836–41. doi: 10.3760/cma.j.issn.2095-4352.2018.09.004 30309408

[12] Moar P, Tandon R. Galectin-9 as a biomarker of disease severity. Cell Immunol. 2021;361:104287. doi: 10.1016/j.cellimm.2021.104287 33494007

[13] Kandel S, Adhikary P, Li G, Cheng K. The TIM3/Gal9 signaling pathway: an emerging target for cancer immunotherapy. Cancer Lett. 2021;510:67–78. doi: 10.1016/j.canlet.2021.04.011 33895262 PMC8168453

[14] Berglundh T, Armitage G, Araujo MG, Avila-Ortiz G, Blanco J, Camargo PM, et al. Peri-implant diseases and conditions: consensus report of workgroup 4 of the 2017 World Workshop on the Classification of Periodontal and Peri-Implant Diseases and Conditions. J Clin Periodontol. 2018;45 Suppl 20:S286–91. doi: 10.1111/jcpe.12957 29926491

[15] Yilihamujiang H, Ni X, Yu M, Dong S, Mei L, Zheng Y, et al. Serum TNF-α level and probing depth as a combined indicator for peri-implant disease. Braz J Med Biol Res. 2024;57:e12989. doi: 10.1590/1414-431X2023e12989 38265340 PMC10802234

[16] Yang B-Y, Deng G-Y, Zhao R-Z, Dai C-Y, Jiang C-Y, Wang X-J, et al. Porous Se@SiO2 nanosphere-coated catheter accelerates prostatic urethra wound healing by modulating macrophage polarization through reactive oxygen species-NF-κB pathway inhibition. Acta Biomater. 2019;88:392–405. doi: 10.1016/j.actbio.2019.02.006 30753941

[17] Chen R, Wang J, Dai X, Wu S, Huang Q, Jiang L, et al. Augmented PFKFB3-mediated glycolysis by interferon-γ promotes inflammatory M1 polarization through the JAK2/STAT1 pathway in local vascular inflammation in Takayasu arteritis. Arthritis Res Ther. 2022;24(1). doi: 10.1186/s13075-022-02960-1 PMC9743547 36510278

[18] Liu L, Stokes JV, Tan W, Pruett SB. An optimized flow cytometry panel for classifying macrophage polarization. J Immunol Methods. 2022;511:113378. doi: 10.1016/j.jim.2022.113378 36265578

[19] Cheng L, Ruan Z. Tim-3 and Tim-4 as the potential targets for antitumor therapy. Hum Vaccin Immunother. 2015;11(10):2458–62. doi: 10.1080/21645515.2015.1056953 26211834 PMC4635934

[20] Solinas C, De Silva P, Bron D, Willard-Gallo K, Sangiolo D. Significance of TIM3 expression in cancer: From biology to the clinic. Semin Oncol. 2019;46(4–5):372–9. doi: 10.1053/j.seminoncol.2019.08.005 31733828

[21] Galarraga-Vinueza ME, Obreja K, Ramanauskaite A, Magini R, Begic A, Sader R, et al. Macrophage polarization in peri-implantitis lesions. Clin Oral Invest. 2020;25(4):2335–44. doi: 10.1007/s00784-020-03556-2 PMC7966129 32886246

[22] Sun J, Huang Q, Li S, Meng F, Li X, Gong X. miR-330-5p/Tim-3 axis regulates macrophage M2 polarization and insulin resistance in diabetes mice. Mol Immunol. 2018;95:107–13. doi: 10.1016/j.molimm.2018.02.006 29433065

[23] Sanz M, Marco Del Castillo A, Jepsen S, Gonzalez-Juanatey JR, D’Aiuto F, Bouchard P, et al. Periodontitis and cardiovascular diseases: consensus report. J Clin Periodontol. 2020;47(3):268–88. doi: 10.1111/jcpe.13189 32011025 PMC7027895

[24] Wang L-X, Zhang S-X, Wu H-J, Rong X-L, Guo J. M2b macrophage polarization and its roles in diseases. J Leukoc Biol. 2019;106(2):345–58. doi: 10.1002/JLB.3RU1018-378RR 30576000 PMC7379745

[25] Kang Z-P, Wang M-X, Wu T-T, Liu D-Y, Wang H-Y, Long J, et al. Curcumin alleviated dextran sulfate sodium-induced colitis by regulating M1/M2 macrophage polarization and TLRs signaling pathway. Evid Based Complement Alternat Med. 2021;2021:3334994. doi: 10.1155/2021/3334994 34567209 PMC8463179

[26] Lv R, Bao Q, Li Y. Regulation of M1‑type and M2‑type macrophage polarization in RAW264.7 cells by Galectin‑9. Mol Med Rep. 2017;16(6):9111–9. doi: 10.3892/mmr.2017.7719 28990062

[27] Xu J, Jiang C, Wang X, Geng M, Peng Y, Guo Y, et al. Upregulated PKM2 in macrophages exacerbates experimental arthritis via STAT1 signaling. J Immunol. 2020;205(1):181–92. doi: 10.4049/jimmunol.1901021 32503893

[28] Zhang J, Hou C, Dou S, Li G, Wang Z, Liu Y, et al. T cell immunoglobulin and mucin domain protein 3 inhibits glycolysis in RAW 264.7 macrophages through Hexokinase 2. Scand J Immunol. 2021;93(2):e12981. doi: 10.1111/sji.12981 33031600

[29] Severino VO, Napimoga MH, de Lima Pereira SA. Expression of IL-6, IL-10, IL-17 and IL-8 in the peri-implant crevicular fluid of patients with peri-implantitis. Arch Oral Biol. 2011;56(8):823–8. doi: 10.1016/j.archoralbio.2011.01.006 21306703

[30] Zheng Z, Ao X, Xie P, Jiang F, Chen W. The biological width around implant. J Prosthodont Res. 2021;65(1):11–8. doi: 10.2186/jpr.jpor_2019_356 32938861

[31] Sculean A, Gruber R, Bosshardt DD. Soft tissue wound healing around teeth and dental implants. J Clin Periodontol. 2014;41 Suppl 15:S6–22. doi: 10.1111/jcpe.12206 24641001

[32] Vignoletti F, Abrahamsson I. Quality of reporting of experimental research in implant dentistry. Critical aspects in design, outcome assessment and model validation. J Clin Periodontol. 2012;39(s12):6–27. doi: 10.1111/j.1600-051x.2011.01830.x 22533944

[33] Galarraga-Vinueza ME, Dohle E, Ramanauskaite A, Al-Maawi S, Obreja K, Magini R, et al. Anti-inflammatory and macrophage polarization effects of Cranberry Proanthocyanidins (PACs) for periodontal and peri-implant disease therapy. J Periodontal Res. 2020;55(6):821–9. doi: 10.1111/jre.12773 32557637

[34] Ozleyen A, Yilmaz YB, Tumer TB. Dataset on the differentiation of THP-1 monocytes to LPS inducible adherent macrophages and their capacity for NO/iNOS signaling. Data Brief. 2021;35:106786. doi: 10.1016/j.dib.2021.106786 33553532 PMC7851796

